# *In vitro *and *in vivo *anticancer properties of a *Calcarea carbonica *derivative complex (M8) treatment in a murine melanoma model

**DOI:** 10.1186/1471-2407-10-113

**Published:** 2010-03-25

**Authors:** Fernando SF Guimarães, Lucas F Andrade, Sharon T Martins, Ana PR Abud, Reginaldo V Sene, Carla Wanderer, Inés Tiscornia, Mariela Bollati-Fogolín, Dorly F Buchi, Edvaldo S Trindade

**Affiliations:** 1 Laboratório de Pesquisa em Células Inflamatórias e Neoplásicas Depto de Biologia Celular, Setor de Ciências Biológicas, Federal University of Paraná, Brazil; 2 Cell Biology Unit (CBU), Institut Pasteur de Montevideo (IPMon), Uruguay

## Abstract

**Background:**

Melanoma is the most aggressive form of skin cancer and the most rapidly expanding cancer in terms of worldwide incidence. Chemotherapeutic approaches to treat melanoma have had only marginal success. Previous studies in mice demonstrated that a high diluted complex derived from *Calcarea carbonica *(M8) stimulated the tumoricidal response of activated lymphocytes against B16F10 melanoma cells *in vitro*.

**Methods:**

Here we describe the *in vitro *inhibition of invasion and the *in vivo *anti-metastatic potential after M8 treatment by inhalation in the B16F10 lung metastasis model.

**Results:**

We found that M8 has at least two functions, acting as both an inhibitor of cancer cell adhesion and invasion and as a perlecan expression antagonist, which are strongly correlated with several metastatic, angiogenic and invasive factors in melanoma tumors.

**Conclusion:**

The findings suggest that this medication is a promising non-toxic therapy candidate by improving the immune response against tumor cells or even induce direct dormancy in malignancies.

## Background

Melanoma represents a significant worldwide public health risk and, from the standpoint of incidence, is the fastest growing of all cancer types. Malignnant melanoma is the most aggressive form of skin cancer, with a mortality rate that has risen about 2% annually since 1960. Although early stage melanoma can be cured surgically, once melanoma metastasizes to major organs (stage IV), it is almost always incurable [1]. There are few chemotherapeutic agents available for treating metastatic melanoma, and those that have been used have all yielded unsatisfactory results. No single chemotherapeutic agent currently offers a response rate greater than 25%, and treatment is invariably accompanied by significant side effects, including myelosuppression, nausea and emesis [2-4].

Malignant transformation could be associated with general enzymatic changes leading to increased proteolytic and fibrinolytic activity in tumor cells. The significance of angiogenesis in tumor development and metastasis is well established, and it was previously confirmed that a significant correlation exists between tumor angiogenesis and the ability of a melanoma to metastasize [5]. It has been observed that heparan sulfate proteoglycan expression, especially perlecan expression, is a prerequisite for melanoma tumor growth and metastasis [6,7].

Immunotherapy remains the subject of intense investigation in both adjuvant and advanced disease settings, and attempts are being made to target melanoma defense mechanisms that blunt the effectiveness of host immune responses [8]. Many natural compounds have been extensively studied to determine a possible anti-tumor effect. Our previous studies have demonstrated that a high diluted natural complex activates macrophages both *in vivo *and *in vitro *[9-11]. Moreover, that natural complex was neither toxic nor mutagenic [12]. Similarly, an improvement of the immune response of treated mice has been demonstrated in studies with Sarcoma-180, including a significant infiltration of lymphoid cells, granulated tissue, fibrosis development around the tumor, sarcoma size reduction and an increase in the number of circulating leukocytes, such as B, NK and CD4^+ ^cells [13]. These results suggest that the medication has a direct or indirect action on hematopoiesis. A subsequent microscopic study of bone marrow cells showed that monocytic lineage (CD11b^+^) and stromal cells (adherent cells) were activated by treatment [14-16]. A previous study with an *in vitro *model showed that a specific high diluted complex derived from *Calcarea carbonica *and associations (here defined as M8 in the Materials and Methods section) activated lymphocytes even without direct contact with macrophages. A co-culture with macrophages and lymphocytes in the presence of M8 promoted stimulation of lymphocytes, resulting in an enhanced tumoricidal performance against a very aggressive lineage of melanoma cells [17].

We previously reported that M8 showed a promising capacity to stimulate immune cells against melanoma cells *in vitro*. In the present work we aimed to study the effects of M8 *in vivo *using a melanoma metastasis mouse model. We assessed blood and bone marrow cells immunophenothyping, tumor histopathology, immunohistochemistry, and ultrastructural analysis.

## Methods

### M8 complex

High diluted natural complexes represent a new form of immunomodulatory therapy and follow Hahnemann's ancient homeopathic techniques for dilution. Mother tinctures were purchased from authorized agencies sanctioned by the Brazilian Health Ministry. These agencies assure the quality (endotoxin free) and physico-chemical composition of their products. Starting from the original mother tincture -- an ethanolic extract in this case -- several dynamizations/succussion (shaking) and serial dilutions in distilled water were performed. The medication used to treat *in vitro *and *in vivo *models was a complex matrix obtained from *Calcarea carbonica *CH5 with associations, comprised of a 10%-20% concentration of each compound obtained after vigorous shaking. In addition to *Calcarea carbonica*, the final solution contained *Aconitum napellus, Arsenicum album, Asa foetida, Conium maculatum, Ipecacuanha, Phosphorus, Rhus tox, Silicea, Sulphur*, and *Thuya occidentalis*, all in decimal dilutions of Hahnemann (dH) in distilled water. The resulting aqueous solution was colorless and odorless, and had 1% alcohol concentration. This complex as well its vehicle (hydroalcoholic solution) used as control were sterilized by filtration through 0.22 μm MILLEX GV Durapore PVDF membranes (Millipore, Billerica, MA, USA), maintained at room temperature and vigorously shaken (succussed) immediately before each treatment. Since all experiments were performed in a double blind and randomized manner, the initial code applied for this studied complex (M8) remains in the Results/Discussion sections of this article.

### Animals

For *in vivo *experiments, 2-3 month old male C57BL/6 mice were purchased from Central Animal House of the Federal University of Paraná (UFPR). All recommendations of the National Law (No. 6.638, November, 5, 1979) for scientific management of animals were followed and the Institutional Animal Care Committee of UFPR approved all related practices. All experiments were performed at least three times in quadruplicate and data analysis was performed in a double-blinded manner. Experiments were carried out at Laboratório de Pesquisa em Células Neoplásicas e Inflamatórias, UFPR, Brazil, and at Cell Biology Unit, IPMon, Uruguay.

### Cell lines

#### B16F10 cells

Murine melanoma cells (B16F10 - ATCC: CRL-6475; mouse melanoma cell), were used for this study. Cells were maintained in DMEM containing 10% FBS, 1 U/mL penicillin, 1 μg/mL streptomycin, and 2.5 μg/mL amphotericin at 37°C in a humidified 5%CO_2 _atmosphere.

#### MxRage 7 cells

Transformed murine embryonic fibroblasts consist of an indicator cell line in which Cre-recombinase is driven by the IFN-inducible Mx1 promoter that, when activated, deletes a stop cassette upstream of the eGFP coding region, resulting in the expression of eGFP. The percentage of eGFP expressing cells, determined by fluorescence activated cell sorting (FACS), accurately correlates to the amount of type I IFN added to the culture [18]. This cell line was maintained at 32°C in a humidified 5% CO_2 _atmosphere, using DMEM (GIBCO, Invitrogen, Carlsbad, CA, USA) supplemented with 10% FBS (GIBCO).

#### HT29

Human colon-rectal cancer cells (ATCC: HTB-38) were stably transfected with the pNF-κB-hrGFP Plasmid from the PathDetect Signal Transduction Pathway *cis*-Reporting Systems Kit (Stratagene) Briefly, subconfluent HT29 cells were transfected with pNF-κB-hrGFP plasmid using Lipofectamine 2000 (Invitrogen) and selected with hygromycin. After two weeks, cells were stimulated for 24 h with a pro-inflammatory cocktail (25 ng/mL TNF-α, 1.25 ng/mL IL-1β and 3.75 ng/mL IFN-γ) and GFP positive cells were sorted with a MoFlo cell sorter (Dako, Carpinteria, CA). This sorted cell line was maintained in RPMI (GIBCO) containing 10% FBS, 1 U/mL penicillin, 1 μg/mL streptomycin at 37°C in a humidified 5% CO_2 _atmosphere.

All *in vitro *experiments were performed at 37°C in a humidified 5% CO_2 _atmosphere for 48 h. All treatments were administered to log-phase growing cells and the different assayed conditions were divided into: a) cells without treatment (control culture conditions, named "Control"); b) cells treated with vehicle (1% hydroalcoholic solution, named "Vehicle"); or c) cells treated with M8 (named "M8"). An initial dose of 20% of M8 treatment was administered to the cells and, after 24 h, a reinforcement dose of 1% was administered according to a previous standard treatment protocol [10,16].

### Experiments on *in vitro *reporter cells (MxRAGE 7 and HT29-pNF-κB-hrGFP)

The reporter cell lines MxRAGE 7 and HT29-pNF-κB-hrGFP are routinely used at CBU (IPMon) to screen natural or synthetic compounds that interfere with type I IFN signaling pathway and/or modulate NF-κB activity. For the IFN assay, exponentially growing MxRage 7 cells were seeded in a 96-well plate and grown for 24 h at 32°C. The cells were then cultured for 48 h in presence or absence of fixed amounts of murine IFN-α11, with or without the vehicle or M8. Finally, cells were harvested using trypsin, resuspended in PBS and stained with 7-AAD to identify dead cells. Ten thousand events were acquired and analyzed on a CyAn™ ADP Flow Cytometer (Dako, Carpinteria, CA) using Summit v4.3 software. For the NF-κB activation assay, exponentially growing HT29 pNF-κB-hrGFP cells were treated cultured for 24 h in absence or presence of 3 ng/mL TNF-α, with or without the vehicle or M8 complex, and the percentage of positive GFP cells and the viability were determined using CyAn™ ADP Flow Cytometer (Dako, Carpinteria, CA) and Summit v4.3 software.

### *In vitro *B16F10 cell experiments

Invasion activity of melanoma cells was assayed in a transwell cell culture chamber as previously described [19], with some modifications. Briefly, polyvinyl-pyrrolidone-free polycarbonate inserts with an 8.0 μm pore size (Corning-Costar, Cambridge, MA, USA) were pre-coated with 5 μg of fibronectin on the reverse side and dried at room temperature. Matrigel (containing laminin, collagen type IV, heparan sulfate proteoglycan and entactin from BD Pharmigen, San Diego, CA, USA) was diluted to 500 μg/mL with cold PBS, applied to the upper surface of the filter (5 μg/filter), and dried at room temperature. Log-phase growing B16F10 cultures were harvested by trypsin treatment, washed twice and re-suspended to give a final concentration of 2.0 × 10^6 ^cel/mL in serum free DMEM medium supplemented with 0.1% bovine serum albumin (BSA, Sigma-Aldrich Chemical Co., St. Louis, MO, USA). Cell suspensions (100 μl) were added to the upper compartment of the insert chamber and incubated for 6 h at 37°C in a humidified 5% CO_2 _atmosphere in the presence or absence (control) of M8 or its vehicle. Cells derived from the different assayed conditions were processed for scanning electron microscopy (SEM). Cell-containing filters were fixed with 2.5% glutaraldehyde (0.1 M cacodylate buffer, pH 7.2), washed with PBS and post-fixed in 1% OsO_4 _for 30 min in the dark at room temperature. After washing, the cells were dehydrated using increasing ethanol concentrations. Filter membranes were CO_2 _critical point dehydrated, metalized and observed using a JEOL JSM-6360 LV SEM.

Cultured B16F10 cells were analyzed by flow cytometry for cadherin, CD74 and 7-Amino-actinomycin D (7-AAD) as viability markers and propidium iodide (PI) as a cell cycle marker. Log-phase growing B16F10 cultures were harvested by trypsin digestion, washed twice and re-suspended in PBS containing 1% FBS. The harvested cells (10^6^) were incubated with anti-CD74/FITC (BD Pharmingen, San Diego, CA, USA) in PBS containing 1% FBS for 30 min and washed three times with PBS. For intracellular CD74 and cadherin, a simultaneous incubation was performed with anti-CD74/FITC (BD Pharmingen) and anti-cadherin/FITC (Vector Labs, Burlingame, CA, USA) antibodies using 0.01% saponin in the incubation buffer. Cells were also stained with 7-AAD (BD Pharmingen) for 5 min to estimate the number of dead cells. Cell cycle was determined as previously described [20]. Log-phase growing B16F10 cultures were detached by trypsin treatment, washed twice with PBS and fixed in chilled 70% ethanol. After centrifugation, the fixed cell pellet was treated with RNAse at a concentration of 50 μg/mL (Sigma-Aldrich Chemical Co., St. Louis, MO, USA) and stained with 50 μg/mL propidium iodide (Sigma-Aldrich) for 10 min at room temperature. For all flow cytometry experiments, ten thousand events were acquired on a FACSCalibur using the CellQuest software (Becton-Dickinson), and data were analyzed using WinMDI 2.9 software.

### B16F10 lung metastasis and *in vivo *treatment

**Figure 1:**
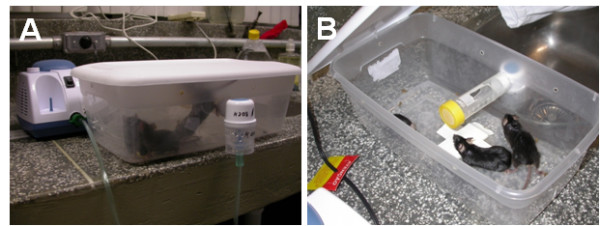
**Inhalation chamber designed to nebulize aqueous compounds for *in vivo *treatment of small rodents, directly distribution compounds to the lung, the specific pathologic site**. The chamber was mounted in a plastic box that was coupled into a micropump nebulizer that was situated at the inlet of the chamber (A), and M8 was nebulized through the inlet into the chamber (B).

B16F10 melanoma cells from a 70-80% confluent monolayer culture were trypsinized, washed and suspended in DMEM. 1 × 10^5 ^cells were then resuspended in 0.1 mL of serum free DMEM and injected intravenously through the tail vein of C57BL/6 mice for the development of lung metastasis. The treatment was started 24 hr after tumor cell inoculation. Animals (n = 9 animals per group) were treated with inhalation of M8 or vehicle twice a day (12-12 hr) for 14 days, or were part of the control group that was not subjected to any treatment. The inhalation chamber was similar to the design previously described [21]. It nebulizes aqueous compounds for *in vivo *treatment of small rodents, aiming to directly distribute compounds to the lung, the specific pathologic site. The chamber was mounted in a plastic box that was coupled to a micropump nebulizer (Inalar - NS, Brazil), which was situated at the inlet of the chamber. M8 or its vehicle was nebulized through the inlet into the chamber (Figure 1). Air was circulated from the chamber outlet through small pores in the box. Following a dose of 10 mL/5 mice/10 min, the mice were removed from the chamber and allowed to equilibrate for 15 min and then replaced in the animal facility until the next dose was administered. After treatment, animals were subjected to euthanasia by an intraperitoneal injection of thiopental, followed by decapitation. Blood from each animal was collected in disodium EDTA vacutainers (BD, San Jose, CA, USA) and was used for immunophenotyping assays. Lungs were chirurgic acquired, placed in Petri dishes with PBS and rapidly analyzed (before histological fixation) with a stereomicroscope for the presence of black metastatic nodules.

### Blood leukocytes and bone marrow cell analysis

For immunophenotyping analyses, blood aliquots containing 10^6 ^leucokytes were incubated for 10 min with PharM Lyse (BD) for red blood cells lysis, followed by an 30 min incubation with anti-CD3/FITC, anti-CD4/PE, anti-CD8/PE, anti-CD11b, anti-CD16/32 (Fc block), anti-CD19/PE, anti-CD45 and anti-Pan-NK/PE. All antibodies were monoclonal antibodies (MAbs) from BD Pharmingen and were diluted in PBS with 1% FBS. After the MAbs binding, cells were washed three times with PBS and analyzed by flow cytometry using a FACSClibur (BD). For each sample, ten thousand events were acquired using the CellQuest software (BD) and data analysis was performed using WinMDI 2.9 software. FITC and PE isotype controls (BD Pharmingen) were used to calibrate the cytometer before the experimental sample acquisition. For bone marrow cells, the protocol was performed as previously described [14]. Briefly, femurs were dissected and cleaned. Epiphyses were removed and the marrow was flushed with DMEM (GIBCO). Cells (10^6^) were fixed with 1% paraformaldehyde, washed, and incubated for 40 min with biotinylated antibodies from a Mouse Lineage Panel Kit (BD, Pharmingen): anti-CD3, anti-B220, anti-Ly6G, anti-TER119, anti-CD11b, anti-CD11c. Samples were then washed with PBS and incubated with 0.5 μg/mL of streptavidin-PE (BD, Pharmingen) in PBS for 30 min and subsequently, cells were acquired and analyzed for blood samples as described. Blood aliquots were also stained with May-Gründ-Giemsa (InstantProv Kit, NewProv, Brazil), allowing for differential counting of basophils, neutrophils, eosinophils and monocytes by light microscopy.

### Lung histopathology, immunohistochemistry, and ultrastructural pathology

After blood sampling, lungs were dissected, washed once with PBS and fixed by immersion in 4% paraformaldehyde in PBS for 1 hr. After washing with PBS, lungs were dehydrated using increasing ethanol concentrations, xylene and embedded in paraffin. For histopathology analysis, 5 μm sections of lungs were deposited on silane coated slides. Sections were deparaffinized in xylene, hydrated in alcohol, and incubated in 3% H_2_O_2 _in methanol to block endogenous peroxidase activity. A Retrievagen A (pH 6.0) kit (BD Pharmingen) was used for antigen unmasking, nonspecific binding was blocked by 1% BSA-PBS solution and possible aldehyde groups were blocked by 50 mM glycine-PBS. Anti-CD11c, anti-GR-1 (BD) and anti-perlecan proteoglycan (Affinity Bio-reagents), as well as the isotype controls (BD Pharmingen), were diluted 1:50 in 0.1% BSA/PBS and incubated on slides in a humidified chamber for 2 hr. After washing with PBS, the respective secondary biotinylated antibody (BD Pharmingen) was added at the same dilution and incubated for 1 hr more, then washed, followed by an incubation step with streptavidin-HSP (BD Pharmingen) for 1 hr. After washing with PBS, MAbs binding was revealed with a DAB Substrate kit (BD Pharmingen). Slides were stained with Giemsa (EMS) and rapidly dehydrated in ethanol, xylene and then coverslips were mounted with entellan. Sections were scanned with a Nikon Eclipse E200 microscope and the areas of the highest immunostaining were imaged at (12.5 × 100)× magnification. CD11c and GR-1 positive spots were counted in 10 chosen fields with highest density. Perlecan expression in tumor zones was quantified by color area measurement by ImageJ software (NIH) to distinguish DAB stain from melanin, which turns color from brown to dark green after Giemsa stain [22]. For conventional histopathology analysis, a periodic acid-Schiff (PAS) stain was performed on the same samples and sections were scanned by a microscope at (12.5 × 40)× or 100× magnification.

For transmission electron microscopy, a rapid protocol was performed as previously described [23]. Briefly, small pieces of lung tissue (1 mm^3^) containing melanoma nodules were fixed for 20-30 min with Karnovsky's fixative (2% glutaraldehyde, 4% paraformaldehyde, 5 mM CaCl_2_, in 0.1 M cacodylate buffer, pH 7.2 - 7.4); they were then washed with the same buffer and post-fixed with 1% osmium tetroxide, 1 mM CaCl_2_, 0.8% potassium ferricyanide in 0.1 M cacodylate buffer (pH 7.4) for 15 minutes and then rinsed twice for 1 min with the same buffer. The lung pieces were dehydrated with acetone 50%, 70%, 90% and 100% (2×) for 3 minutes each. The tissues were transferred to bean capsules containing 90% acetone and infiltrated in epoxy resin (Epon)/acetone solution (1:1) for 2 hr, then in pure Epon for 4 hr and, lastly, embedded in a new Epon solution overnight. Polymerization was carried out for 48 hr at 60°C. Ultra-thin sections were stained with aqueous uranyl acetate for 15 min and with lead citrate for 2 min. Samples were visualized with a Jeol JEM 1011 transmission electron microscope. A GATAN CCD camera and GATAN digital micrograph software were used to obtain the digital images.

### Micrographs and statistics analysis

Micrographs obtained from immunohistochemistry and electron microscopy were analyzed by ImageJ software (NIH) to obtain mean/pixel values from the specific threshold of transwell insert pores or IHC chromogen. Data obtained from assays were transformed to conform to a normal distribution using the equation *transformed data *= . Statistical significance of transformed data was determined using a one-way analysis of variance (ANOVA), followed by Tukey post test. Statistical significance is presented as either P < 0.05 (*) or P < 0.01 (**). Data are representative of three independent experiments performed in triplicate.

## Results

### M8 *in vitro *effects on B16F10 cells

**Figure 2:**
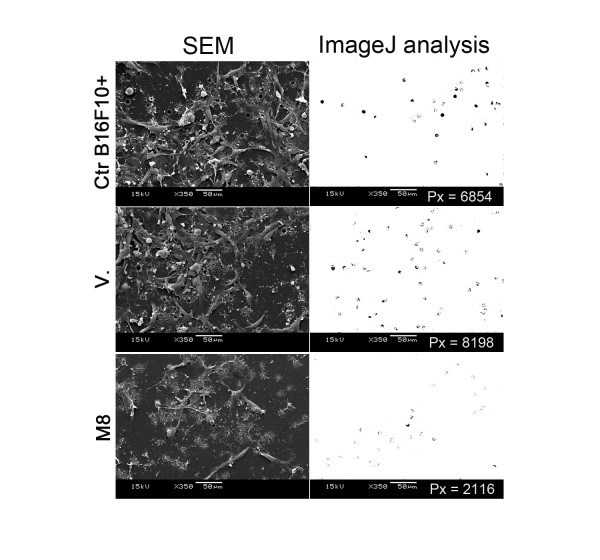
***In vitro *treatment of B16F10 cells**. Untreated cells (Ctr. B16F10+), vehicle treated cells (V) and M8 treated cells (M8), respectively. Scanning electron micrographs (SEM) of invasion assay on fibronectin/matrigel-coated transwell inserts with 8 μm pores. Original micrographs were analyzed by ImageJ software and the total area of 8 μm pores was evaluated in pixels (Px). Black density of pores is directly proportional to matrigel wall degradation by B16F10 cells. ImageJ analysis of pores shows the loss of adhesion of B16F10 cells, accompanied by a decrease in cell number on the matrigel substrate, as well as the permanence of non-degraded matrigel obstructing the pores in the cells treated with M8, diminishing the number of exposed pores.

First, we wanted to address the *in vitro *effect of M8 treatment on B16F10 cells. After 48 h exposure, viability, cell cycle, protein expression associated with tumor process and adhesion/invasion in matrigel were analyzed. Cell viability, assessed by 7-AAD stain, did not show significant differences between control, vehicle and M8 (data not shown). Cell cycle was evaluated and there was a clear trend toward a decrease in the number of cells in S and G2 phases; however, the data did not reach statistical significance (p = 0.08) (data not shown). Flow cytometry analysis of the expression pattern of some key proteins linked with the tumoral process showed no differences in the intracellular expression of E-cadherin or intracellular/extracellular expression of CD74 (data not shown). However, when the effect of M8 treatment in adhesion and invasion in matrigel was assessed, a promising result was observed. Electron microscopy analysis of the invasion assay on fibronectin/matrigel coated in transwell inserts with 8 μm pores showed a decrease in B16F10 cell adhesion in matrigel that was associated with a decrease in matrigel degradation after M8 treatment (Figure 2). ImageJ analysis of the pores clearly shows a loss of adhesion of B16F10 cells, accompanied by a decrease in the number of cells on the matrigel subtract. When cells were treated with M8, there was more non-degraded matrigel obstructing the pores, leading to a diminished number of exposed pores. These data suggest that M8 decreases cellular invasion.

### *In vivo *evaluation of M8 treatment in lung metastasis

**Figure 3:**
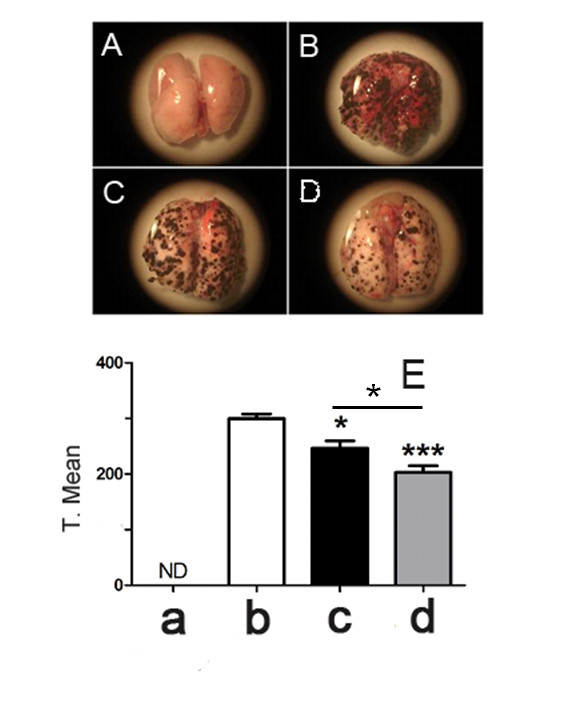
**Lung metastasis in C57BL/6 mice induced from B16F10 tail vein injection**. **Figure A**: Normal lung. **Figure B, C and D**: A mouse with B16F10 lung metastasis, untreated, vehicle treated or M8 treated, respectively. **Figure E: a **= normal lung; **b **= untreated lung with B16F10 metastasis; **c **= lung with B16F10 metastasis treated with vehicle; **d **= lung with B16F10 metastasis treated with M8. Treatment caused a statistically significant decrease in metastasis (***P < 0.001) and significantly decreased metastasis as compared with vehicle-treated animals (*P < 0.05). Y axis of graph = transformed mean after normal distribution using the equation to transform data, .

Taking into account the promising results obtained *in vitro*, the next step was to evaluate the *in vivo *effect of M8 in a lung metastasis model. C57BL/6 mice were injected intravenously with B16F10 cells to develop lung metastasis. The treatment was started 24 hr after tumor cell inoculation and the animals were treated with M8 or vehicle twice a day for 14 days. A decrease in the number of tumoral nodules was observed in lungs after M8 treatment (Figure 3A-D). This observation was confirmed by the statistical analysis of the number of tumor nodules, which showed that the difference between the control and the M8-treated mice was significant (***P < 0.001) (Figure 3E).

**Figure 4:**
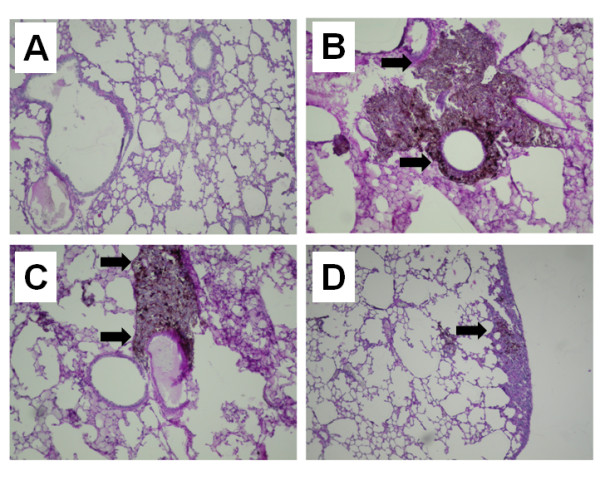
**Histopathology analysis of normal lung (A), lung metastasis from untreated mice, control (B), lung metastasis from mice treated with the vehicle (C) and lung metastasis from mice treated with M8 (D)**. A significant difference was observed in tumor nodule pattern distribution, concentration and area. After M8 treatment (D), tumor nodules (arrows) were smaller and organized in a predominantly peripheral distribution, whereas in the control and vehicle groups the nodules were larger and distributed in the lung parenchyma (B and C, respectively).

Histopathology analysis showed a difference in tumor nodule pattern distribution, concentration and area in lung histology sections. From a structural point of view, it was observed that melanoma cells with poliedric morphology with a great amount of melanin content as cytoplasm granules or in a perinuclear distribution. Additionally, aberrant nodular proliferation in bronchoalveolar regions, characteristic of epithelial melanoma, was observed (Figure 4B and 4C, respectively). After M8 treatment (Figure 4D), tumor nodules were decreased and organized in a predominantly peripheral distribution, whereas, in control and vehicle groups, the nodules were larger and distributed in the lung parenchyma.

**Figure 5:**
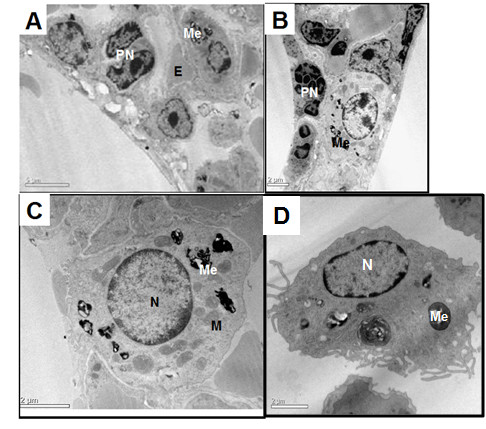
**Ultrastructural overview by Transmission Electron Microscopy - Images of lungs with melanoma nodules**. **A**: Lung cells in panoramic view, with nucleus (N), erythrocytes (E), pyknotic nucleus (PN), melanosomes (Me); **B**: Lung in a panoramic view with an evident tumor cell containing a big nucleus, melanosomes (Me) and many mitochondria; **C**: more details of a tumor cell, including the morphologic characteristics of intense metabolism, a big euchromatic nucleus, many mitochondria (Mi) and melanosomes. **D**: an alveolar macrophage with an euchromatic nucleus (N); many organelles, such as the endoplasmic reticulum and mitochondria, endocytic vacuoles, and internalized melanosomes (Me); and a surface covered with many cellular projections.

An ultrastructural overview by transmission electron microscopy showed a characteristic pulmonary parenchyma (Figure 5), in which the presence of tumor cells with large amounts of very dense and black cytoplasmic pigment granules (melanosomes) is obvious. Surrounding these cells are many dying parenchyma cells with pyknotic nucleus (Figure 5A and 5B), characteristic of apoptotic cells. Figure 5C shows a tumor cell at higher magnification, characterized by melanosome granules and increased metabolic activity, evidenced by a euchromatic nucleus and many mitochondria. Figure 5D shows a macrophage with internalized melanosomes, evidence of the phagocytosis of tumor cell products.

### Peripheral blood and bone marrow cell subpopulation analysis after M8 treatment of mice with lung metastasis

**Figure 6:**
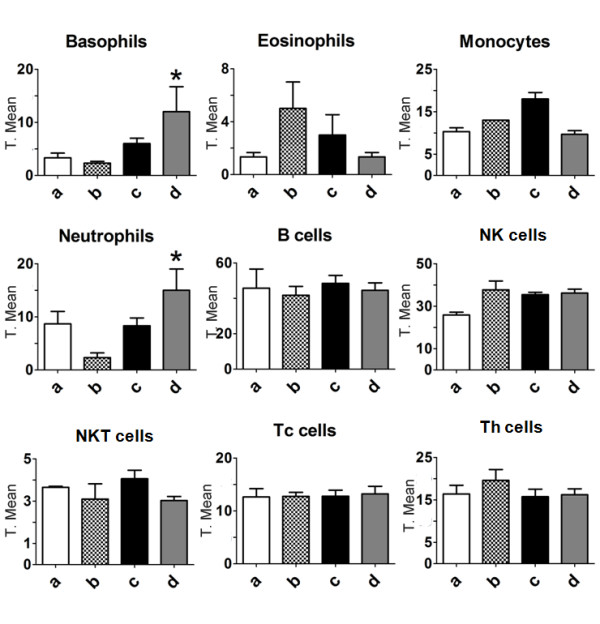
**Blood leukocyte phenotyping**. May-Gründ-Giemsa stain was performed to differentially count basophils, neutrophils, eosinophils and monocytes by light microscopy. MABs for CD3/CD4, CD3/CD8, CD3/CD19, and CD3/Dx5 were used to evaluate B (CD3^-^CD19^+^), NK (CD3^-^Dx5^+^), NKT (CD3^+^Dx5^+^), Th (CD3^+^CD4^+^), and Tc (CD3^+^CD8^+^) lymphocytes. **a**: normal mice; **b**: untreated mice with B16F10 lung metastasis; **c**: vehicle-treated mice with B16F10 lung metastasis and **d**: M8-treated mice with B16F10 lung metastasis. Treatment caused a statistically significant increase in the number of blood basophils and neutrophils (*P < 0.05). Y axis of graphs = transformed mean after normal distribution using the equation to transform data, .

**Figure 7:**
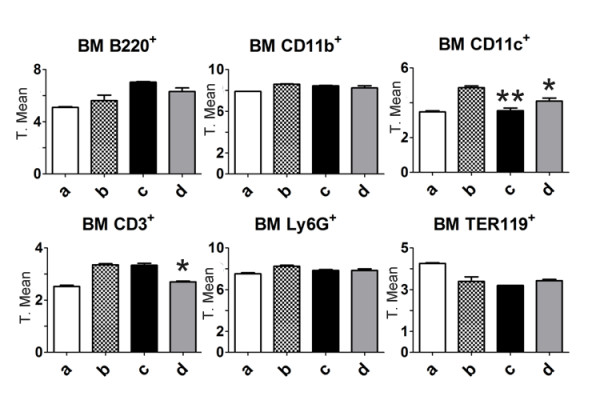
**Bone marrow cell phenotyping**. MABs for CD3, CD11b, CD11c, B220, Ly6G and TER119 were used to evaluate T precursor cells (CD3^+^), monocyte precursors (CD11b^+^), dendritic cell precursors (CD11c^+^), granulocyte precursors (Ly6G^+^) and erythrocyte precursors (TER119). **a**: normal mice; **b**: untreated mice with B16F10 lung metastasis; **c**: vehicle-treated mice with B16F10 lung metastasis and **d**: M8-treated mice with B16F10 lung metastasis. Most markers showed a normalization tendency, i.e. the values from treated mice were similar to the values from normal mice. Y axis of graphs = transformed mean after normal distribution using the equation, to transform data, .

In order to determine whether the melanoma regression was mediated by immune cells, different subpopulations were quantified from blood and bone marrow derived from mice with lung metastasis. Blood leukocytes analysis from M8 treated mice showed a significant increase in basophils and neutrophils concentrations, but not in eosinophils or monocytes (Figure 6). After lymphocyte immunophenotyping analyses, no significant differences were found in B (CD3^-^CD19^+^), NK (CD3^-^Dx5^+^), NKT (CD3^+^Dx5^+^), Tc (CD8^+^), or Th (CD4^+^) lymphocytes in circulating blood (Figure 6). On the other hand, cells from bone marrow showed differences in the concentration of CD3 and CD11c positive cells, but not in the concentration of B220, CD11b, Ly6G and TER119 positive cells (Figure 7).

### Lung immunohistopathology

**Figure 8:**
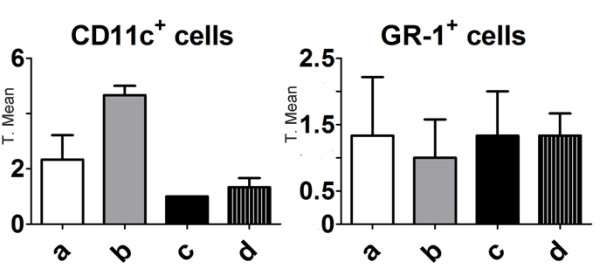
**Immunohistochemistry quantification of CD11c (monocytic/dendritic cell marker) and GR-1 (granulocytic marker) cells**. **a**: normal mice; **b**: untreated mice with B16F10 lung metastasis; **c**: vehicle-treated mice with B16F10 lung metastasis and **d**: M8-treated with B16F10 lung metastasis. No statistically significant differences were found after developing with HSP/TMB (blue) and counting positive cells. Y axis of graph = transformed mean after normal distribution using the equation to transform data, .

**Figure 9:**
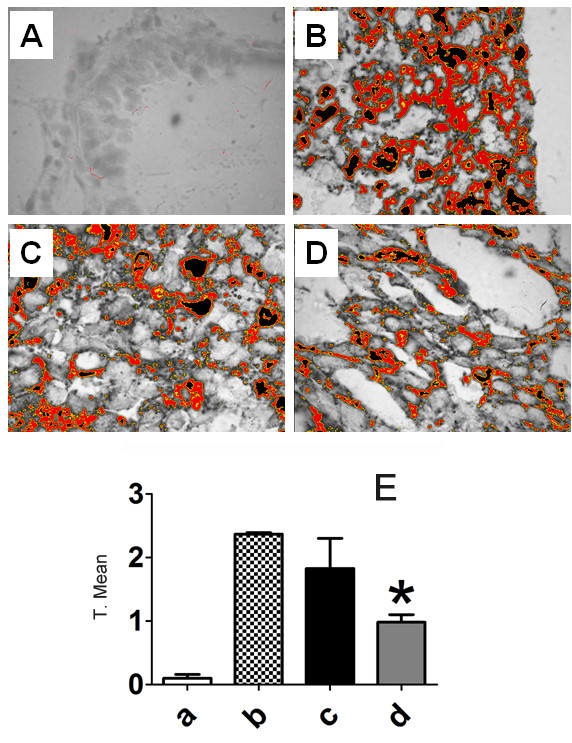
**Perlecan quantification in metastatic nodules by immunohistochemistry**. Staining melanocytic samples with Giemsa results in a uniform dark green/blue coloration of melanin that is easily distinguishable from the DAB precipitate of the HSP reaction. DAB-specific threshold selection from selected tumor areas was performed by ImageJ software (red/orange selection in **Figures A, B, C **and **D**), and total selected area was quantified and statistically analyzed (**E**). **a**: normal mice; **b**: untreated mice with B16F10 lung metastasis; **c**: vehicle-treated mice with B16F10 lung metastasis and **d**: M8-treated mice with B16F10 lung metastasis. M8 treatment resulted in a statistically significant decrease in heparan sulfate proteoglycan expression. **A **= Normal lung; **B **= B16F10 positive control; **C **= Vehicle treatment; **D **= M8 treatment. Y axis of graphs = transformed mean after normal distribution using the equation to transform data, .

The next step was to evaluate whether M8 treatment could affect lung-infiltrated or resident granulocytes and macrophages/dendritic cells. The concentration of those cell populations were estimated by cell counting on immunohistochemistry slides. Although the number of blood granulocytes (basophils and neutrophils) was increased after treatment, no significant differences were found in lung peritumoral granulocyte (GR-1^+^) or macrophages/dendritic cell (CD11c^+^) number (Figure 8). Lung samples were also analyzed by immunohistochemistry for perlecan expression, a heparan sulfate proteoglycan. After M8 treatment, perlecan expression was significantly down regulated in tumor nodules (Figure 9A-D). This difference was more evident after quantification by ImageJ (Figure 9E).

## Discussion

Tumor cells use multiple mechanisms to escape detection and elimination by the immune system, prompting the development of chemotherapeutic drugs that harness both humoral and cellular immunity to target malignant cells. There is little basic research on the effectiveness of alternative and complementary therapies in cancer, and the few studies that have been performed were limited in scope. Beside this, immunostimulation by natural products has been attempted in various animal models and in human cancer patients as an adjunct to chemotherapy [24-26].

A high diluted complex treatment in macrophages has been shown to suppress previously elevated levels of tumor necrosis factor-α (TNF-α), increase the activity of NADPH oxidase and the expression of inducible nitric oxide synthase (iNOS), and induce differential gene expression [9-11]. Many studies have demonstrated the role of different high diluted complexes in cancer therapy immunomodulation [13,17,27,28]. Regarding M8 (*Calcarea carbonica *and associations), our previous results showed that *in vitro *treatment significantly increased macrophages/lymphocyte interaction and effectiveness against melanoma cells [17]. Extending these previous data to the *in vivo *immunotherapeutic effect of this compound, we now demonstrate that M8 therapy prevents tumor growth and metastasis.

Progression of melanoma and other malignant cancers involves cellular changes such as the loss of E-cadherin expression and the gain of CD74 expression, which confer cell motility and immunologic escape, respectively [29,30]. However, while both molecules were analyzed in the present study, no differences were found after M8 treatment (data not shown). Despite this finding, scanning electron micrographs analysis clearly showed the loss of adhesion of B16F10 cells, demonstrated by a decreased cell number on the matrigel subtract, and the permanence of non-degraded matrigel obstructing the pores in the cells treated with M8, leading to a diminished number of exposed pores (Figure 2).

Since interferon type I based immunostimulation has been studied as an effective immunotherapy for melanoma cases [31], we further investigated the *in vitro *effect of M8 on type I IFN activity using MxRage reporter cells [18]. When these cells were exposed to M8, no agonist or antagonism capacity was observed (Additional file 1: Supplementary Figure A). Moreover, NF-κB has been reported to increase tumourigenesis by promoting anti-apoptotic activity, chemotherapy resistance, the expression of positive cell cycle regulators and the expression of other survival factors [32]. However, no agonistic or antagonistic NF-κB activation was found after M8 treatment in the HT29 reporter model (Additional file 1: Supplementary Figure B).

On the other hand, lung metastasis regression was observed *in vivo *after M8 treatment (Figure 3 and 4). In order to evaluate the mechanism of action of M8 *in vivo*, the level of immune cells was analyzed. Previous studies have shown that this category of medicament increases blood lymphocytes such as TCD4, TCD8 and NK, which are associated with tumor regression in an albino Swiss mice/Sar-180 model [13]. In a C57BL/6 mice/B16F10 model, there was no significant difference in lymphocyte level from blood and bone marrow (Figure 6 and 7). However, the difference between the results obtained with the C57BL/6 strain and albino Swiss strain may be because the first mouse model has an intrinsic and well established innate and Th1 response that limits its use immunological questions [33]. On the other hand, it is the most used murine model to simulate metastatic melanoma because it reflects several characteristics of human metastatic melanoma, and because B16F10 cell inoculation is only compatible with this strain [34]. An increase in the number of circulating basophils and neutrophils was observed after M8 treatment. Basophils and neutrophils have been recently appointed as promissory innate cells to be targeted for anti-cancer treatment because are the first cells able to detect tissue abnormalities as tumor necrosis or growth damages tissue [35]. There was no significant difference in bone marrow leukocytes number, but there was a clear tendency for CD4 and CD11c markers (Figure 7) to be close to the levels seen in normal mice, demonstrating a "normalization" of bone marrow cell production.

This study provides some information to clarify the mechanism by which M8 treatment affects melanoma metastasis in this model. We observed a decrease in perlecan expression and a direct inhibition of cancer cell adhesion and invasion (Figure 2 and 9). Perlecan is an important component of basement matrix and its expression correlates strongly with the expression of several metastatic, angiogenic and invasive factors in tumor cells, particularly melanoma cells. Tumor-derived perlecan is distributed throughout its matrix and creates a microenvironment that favors neovascularization, tumor growth and invasion [36,37]. Perlecan has been identified as a potential therapeutic target for the treatment of metastatic cancer because it is a necessary molecule for tumor vessels' structural integrity and forms the major storage site of neovascularization factor FGF-2 [7,38]. Despite this, the inhibition of adhesion of extracellular matrix molecules in melanoma cells causes cell rounding, loss of survival and apoptosis [39]. This result is corroborated by the invasion assay, which showed a loss of adhesion of B16F10 cells to the Matrigel substrate because of a decrease in the ECM proteolysis ability of the cells (Figure 2).

## Conclusion

Taken together, these results may explain the possible tumor cell dormancy and decrease in tumor nodule number and volume caused by M8 treatment. These findings suggest that M8 is a promising therapy that may improve the innate immune response against tumor cells, decreasing the cell adhesion or invasion of malignant cells or even inducing direct dormancy in malignancies. Further studies are necessary to clarify the precise and detailed mechanism of M8 treatment in anti-tumor effects in melanoma and its use as combination-therapy candidate used in addition with conventional medicines.

## Competing interests

The authors declare that they have no competing interests.

## Authors' contributions

FSFG designed and performed all experiments, analysis and drafted the manuscript. RS, IT and MBF collaborated on cell culture and *in vitro *assays. LAF, SHM, APRB and CW collaborated on cell culture and *in vivo *assays. DFB and EST designed and supervised all experiments and manuscript writing. All authors have read and approved the final manuscript.

## Pre-publication history

The pre-publication history for this paper can be accessed here:

http://www.biomedcentral.com/1471-2407/10/113/prepub

## Supplementary Material

Additional file 1***In *vitro type I IFN and NF-κB assays**. In order to verify the possible influence of M8 treatment in type I IFN and/or NF-κB signaling, a preliminary screening was performed. Quantitative examination of M8 treated Mx-RAGE and HT29 cells by flow cytometry showed no statistically significant differences in type I IFN and/or NF-κB activity, respectively (Supplementary Figures A and B, respectively). **Supplementary Figure A**: M8 *in vitro *treatment of MxRage cells, a reporter cell line to evaluate type I IFN activity. No significant differences were found in GFP expression during the screening. Both negative and positive circumstances were evaluated for the ability to increase IFN production (compound alone) and the ability to decrease IFN production (compound plus IFN-α). GFP expression was evaluated by flow cytometry analysis of treated cells. No differences in cell viability were detected by 7-AAD stain. **Supplementary Figure B**: The HT29-pNF-κB-hrGFP reporter cell line was used to evaluate the activation of NF-κB after *in vitro *treatment with M8. No significant differences were found in NF-κB activation after treatment. Both negative and positive scenarios were evaluated for the ability to activate NF-κB (compound alone) and the ability to decrease NF-κB activation (compound plus TNF-α). GFP expression was evaluated by flow cytometry of treated cells. No differences in cell viability were detected by 7-AAD stain. Y axis of graphs = transformed GFP mean after normal distribution using the equation to transform data, .Click here for file
