# Supplementary figures and images for: In vitro and in vivo anticancer properties of a Calcarea carbonica derivative complex (M8) treatment in a murine melanoma model
Source: BMC Cancer. 2010 Mar 25;10:113. doi: 10.1186/1471-2407-10-113 (PMC2859384; doi:10.1186/1471-2407-10-113)

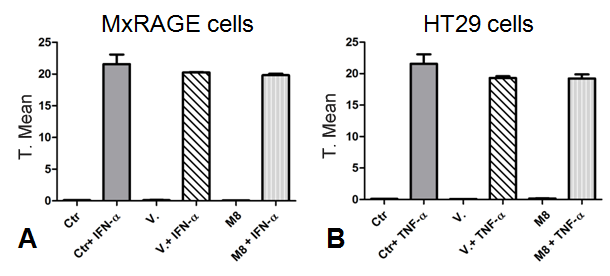

Supplement: Additional file 1 — In vitro type I IFN and NF-κB assays. In order to verify the possible influence of M8 treatment in type I IFN and/or NF-κB signaling, a preliminary screening was performed. Quantitative examination of M8 treated Mx-RAGE and HT29 cells by flow cytometry showed no statistically significant differences in type I IFN and/or NF-κB activity, respectively (Supplementary Figures A and B, respectively). Supplementary Figure A: M8 in vitro treatment of MxRage cells, a reporter cell line to evaluate type I IFN activity. No significant differences were found in GFP expression during the screening. Both negative and positive circumstances were evaluated for the ability to increase IFN production (compound alone) and the ability to decrease IFN production (compound plus IFN-α). GFP expression was evaluated by flow cytometry analysis of treated cells. No differences in cell viability were detected by 7-AAD stain. Supplementary Figure B: The HT29-pNF-κB-hrGFP reporter cell line was used to evaluate the activation of NF-κB after in vitro treatment with M8. No significant differences were found in NF-κB activation after treatment. Both negative and positive scenarios were evaluated for the ability to activate NF-κB (compound alone) and the ability to decrease NF-κB activation (compound plus TNF-α). GFP expression was evaluated by flow cytometry of treated cells. No differences in cell viability were detected by 7-AAD stain. Y axis of graphs = transformed GFP mean after normal distribution using the equation to transform data, . [file 1471-2407-10-113-S1.TIFF]
